# Microsurgical reconstruction of the heel: evaluation and decision-making recommendations based on a case series

**DOI:** 10.1080/23320885.2025.2556491

**Published:** 2025-09-05

**Authors:** Federico Ziani, Corrado Rubino, Silvia Rampazzo, Matilde Tettamanzi, Giovanni Arrica, Ilaria Ginatempo, Claudia Trignano, Fabio Santanelli di Pompeo, Michail Sorotos, Emilio Trignano

**Affiliations:** a Plastic Surgery Unit, University Hospital Trust of Sassari, Sassari, Italy; b Department of Medicine, Surgery and Pharmacy, University of Sassari, Sassari, Italy; c Department of Biomedical Sciences, University of Sassari, Sassari, Italy; d Department of Neuroscience, Mental Health, and Sense Organs, Faculty of Medicine and Psychology, Sant’ Andrea Hospital, Sapienza University of Rome, Rome, Italy

**Keywords:** Skin wound healing and aesthetic outcomes, heel free flap reconstruction, heel defect, microsurgery, trauma

## Abstract

Reconstruction of the calcaneal region presents unique challenges due to its complex anatomy and critical weight-bearing function. This retrospective study aims to report our experience in cases of calcaneal defects repaired with various free flap types, and to evaluate the long-term outcomes of the reconstructions. We retrospectively reviewed 25 patients who underwent microsurgical free flap reconstruction for calcaneal defects between January 1997 and March 2022. Demographics, defect characteristics, surgical techniques, and outcomes were analyzed. Out of the 25 cases, 18 patients (72%) were male, with a median age of 55 years. Successful reconstructions were achieved in 24 patients (96%) using various free flap types: ALT flap (8cases), Parascapular flap (4 cases), Gracilis flap (5 cases), TAP flap (5 cases), Radial forearm flap (2 cases), and in one case, deep circumflex iliac artery (DCIA) osteocutaneous flap. These reconstructions demonstrated good functional recovery and minimal complications. Only one case (4%) experienced flap failure due to venous thrombosis, necessitating revision surgery without success. The most frequent complication was the sliding (6 cases) due to the excessive bulk which was addressed with revision surgery. The average follow-up period was 8 months, with patients regaining satisfactory ambulation and improved quality of life. Based on our results, microsurgical free flap reconstruction has proven to be a valuable technique for addressing calcaneal defects and, according to many Authors, it offers a high success rate and favorable long-term outcomes. In selected cases, revision surgery may be necessary to address bulk-related issues.

## Introduction

Heel reconstruction after trauma, trophic ulcers, burns and tumors resection represents one of the most important challenges in plastic surgery, both for its functional and aesthetic importance. The bodyweight is transmitted for 80% by the heel calcaneal complex [1] and the heel pad acts like a shock absorbing system, for this reason the region is covered by firm, anti-friction plantar fascia, and thicker stratum corneum skin. Being the most important weight-bearing region of the foot, an inappropriate reconstruction in this area can cause gait and walking deficits [2,3].

The reconstruction of the calcaneal region requires the use of soft tissue that can withstand body weight and provide sensation to prevent pressure injuries [4], furthermore it has to provide the right amount of skin, subcutaneous tissue and, in some cases, if bone is missing, it should be replaced. The heel region encompasses several anatomically distinct areas, each presenting unique functional requirements. The plantar heel, as the primary weight-bearing area, is characterized by thick, glabrous skin and a dense fibrofatty pad, providing essential cushioning and shock absorption. Reconstruction in this area necessitates durable tissue capable of withstanding significant pressure and shear forces. In contrast, the posterior heel, located at the back, plays a lesser role in weight-bearing but is crucial for the attachment of the Achilles tendon, where reconstruction focuses on creating stable coverage to protect the tendon and ensure proper function. The medial and lateral heel regions, found on the inner and outer sides, experience varying degrees of pressure and friction, especially during ambulation; therefore, reconstructive efforts in these areas aim to restore contour and provide skin that can endure movement and minor pressure. Finally, the Achilles tendon area encompasses the tendon and its insertion, where reconstruction must offer robust coverage to protect the tendon and facilitate its gliding function.In literature, there are many reconstructive possibilities such as local flaps [5] or free flaps.

The purpose of this paper is to report our experience in calcaneal region reconstruction with free microsurgical flaps and to evaluate their functional outcomes.

## Methods

We retrospectively evaluated a cohort of 25 patients who underwent calcaneal region reconstruction between January 1997 and March 2022. All procedures were performed by two senior surgeons (ET, CR) at the plastic surgery Unit of the University of Sassari (Italy) and at China Medical University Hospital of Taichung (Taiwan). The patient’s medical records were evaluated to obtain demographic data, etiology and characteristics of the defects, type of flap used for the reconstruction, recipient vessels and type of anastomosis. Early postoperative complications, such as hematoma, infection, seroma and flap loss were also recorded. Flap monitoring was performed using traditional flap monitoring based on careful periodic clinical observation. This involved evaluating the color, turgor, temperature, capillary refill, and pin prick at specific time intervals [6].

In cases where flap viability was at risk, techniques such as pre-tie sutures and serial delayed closure [7], were considered to enhance stability and reduce tension, particularly in high-stress areas of the heel.

Furthermore, late complications such as scar contracture, sliding, neuroma and excessive bulk were reviewed, as long as the need of secondary procedures to treat them. The functional recovery after reconstruction was evaluated in terms of the ability to walk without support and wear shoes.

## Results

From January 1997 to March 2022, twenty-five patients underwent soft tissues and bone heel reconstruction with osteomuscular or fasciocutaneous free flaps. Patients’ data and relative type of reconstructions, etiology and defect are described in Table 1. The group consisted of 18 male (72%) and 7 female (28%) patients with a median age of 55 years (range 4 to 80 years). In our case series, indications for heel reconstruction were soft tissue defects (24 cases) and soft tissues combined with bone defects (1 case) involving the calcaneus resulting from trauma (72%), skin cancer resection (16%) and diabetic ulcer (12%). The weight bearing area was involved in 18 cases, while the posterior calcaneal region was affected in the remaining cases. The defect area ranged from 3 cm × 4 cm to cm 13 × 9 cm. In our case series the free flaps used for calcaneal region reconstruction were ALT flap (8 cases), parascapular flap (4 cases), gracilis flap (5 cases), TAP flap (5 cases), deep circumflex iliac artery (DCIA) osteocutaneous flap (1 case) and forearm flap (2 cases). Flap selection was made according to specific location of the damage, range of skin and soft tissue or bone defect. Recipient arteries include posterior tibial artery in 20 patients and the medial plantar artery in 5 patients. In 24 cases end-to-side anastomosis were performed, in one case of Iliac crest flap surgeons performed a t-shaped anastomosis. The donor area was directly sutured or covered with skin grafts.

**Table 1.** Patients data.

Cases	Ageand sex	Etiology	Size of defect	Soft tissue/bone defect	Weight/non-weight bearing area	Type of flap	Recipient vessels	Complications
1	45F	Trauma	8 × 5 cm	ST	WB	ALT flap	Posterior Tibial artery	Excessive Bulk Sliding
2	36M	Trauma	9 × 4 cm	ST	WB	ALT flap	Posterior Tibial artery	Excessive Bulk Sliding
3	37M	Trauma	9 × 6 cm	ST	NWB	Parascapular flap	Posterior Tibial artery	None
4	58M	Trauma	8 × 6 cm	ST	NWB	Gracilis flap + SG	Medial plantar artery	None
5	29M	Trauma	11 × 4 cm	ST	WB	TAP flap	Posterior Tibial artery	None
6	68M	Trauma	9 × 4 cm	ST	NWB	TAP flap	Posterior Tibial artery	None
7	71M	Diabetic Ulcer	8 × 4 cm	ST	WB	TAP flap	Posterior Tibial artery	None
8	43M	Trauma	6 × 3 cm	ST	WB	Parascapular flap	Posterior Tibial artery	None
9	65M	Trauma	5 × 4 cm	ST	WB	ALT flap	Posterior Tibial artery	Excessive Bulk Sliding
10	34F	Trauma	5 × 3 cm	ST	WB	Parascapular flap	Posterior Tibial artery	Hematoma
11	4M	Trauma	3 × 4 cm	ST	WB	ALT flap	Posterior Tibial artery	Scar contracture
12	67F	Trauma	6 × 3 cm	ST	NWB	Parascapular flap	Posterior Tibial artery	Scar contracture
13	34F	Trauma	6 × 4 cm	ST+B	WB	DCIA flap	Posterior Tibial artery	None
14	16M	Trauma	13 × 9 cm	ST	WB	ALT flap	Posterior Tibial artery	Excessive Bulk Sliding
15	51M	Trauma	11 × 4 cm	ST	NWB	TAP flap	Posterior Tibial artery	Hematoma
16	61M	Trauma	8 × 4 cm	ST	WB	Gracilis flap + SG	Medial Plantar artery	Infection
17	55M	Diabetic Ulcer	12 × 6 cm	ST	NWB	TAP flap	Posterior Tibial artery	None
18	80M	Diabetic Ulcer	8 × 4 cm	ST	WB	Gracilis flap	Medial Plantar artery	None
19	42M	Trauma	7 × 4 cm	ST	NWB	Gracilis flap	Medial Plantar artery	None
20	54F	Trauma	9 × 5 cm	ST	WB	Gracilis flap	Medial Plantar artery	Neuroma Ulcers
21	78M	Skin Cancer	3 × 4 cm	ST	WB	Forearm flap	Posterior Tibial Artery	None
22	80M	Skin Cancer	3.5 × 5 cm	ST	WB	Forearm flap	Posterior Tibial Artery	None
23	56F	Skin Cancer	3 × 5 cm	ST	WB	ALT flap	Posterior Tibial Artery	Excessive Bulk Sliding
24	62F	Skin Cancer	4 × 3 cm	ST	WB	ALT flap	Posterior tibial Artery	Excessive Bulk Sliding
25	43M	Skin cancer	11 × 5 cm	ST	WB	ALT flap	Posterior Tibial Artery	Failure

M, Male; F, Female; ST, Soft tissue; B, Bone defect; WB, Weight bearing area; NWB, Not Weight bearing area; SG, Sking graft; ALT flap, Anterolateral tight flap; DCIA flap, Deep circumflex iliac artery flap; TAP flap, Thoracodorsal artery perforator flap.

Incidence of complications is reported in Table 2. Local infection was observed only in one case of gracilis flap: the patient was treated with e.v. antibiotic therapy that led to infection resolution. Two patients suffered from early postoperative hematoma, which was successfully drained in both cases. Flap loss occurred in one patient (28%) due to venous thrombosis; revision of the anastomosis was performed but without success. Therefore, we proceeded to cover the loss of substance with dermal substitute and skin graft.

**Table 2.** Complications.

Complications	Number of cases	Incidence
Hematoma	2	8%
Wound Infection	1	4%
Scar contracture	2	8%
Excessive bulk	6	24%
Sliding	6	24%
Neuroma	1	4%
Ulcer	1	4%
Failure	1	4%

The most frequent complication was the sliding (6 cases) due to excessive flap bulk. Two patients (8%) developed scar contracture, which were corrected with z-plasties. Six patients (24%) required a secondary procedure to reduce flap bulk; all were ALT flaps.

These revisions successfully improved both footwear adaptability and patient comfort during ambulation.

Despite these complications, donor sites healed without significant morbidity. At a mean follow-up of 8 months, all patients—except one who required orthopedic shoes—were able to walk pain-free and wear standard footwear. Additionally, none of the patients experienced late complications such as recurrent ulceration, progressive flap deterioration, or functional impairment. The patients who underwent secondary debulking procedures reported increased mobility and comfort post-revision, further supporting the effectiveness of flap refinement in optimizing functional outcomes.

## Case reports

### Case 1 – free iliac crest flap

**Figure 1.**
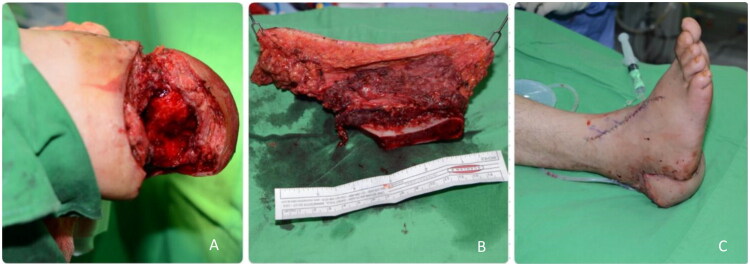
(A) Preoperative picture of the defect. (B) The iliac crest flap is isolated. (C) Immediate post-operative picture.

A 34-year-old female suffered an avulsion injury of the right foot following a motorbike accident, resulting in calcaneal bone and soft tissue loss (6 × 4 cm) (Figure 1A). A DCIA flap was used to reconstruct the defect, with an iliac bone segment replacing the missing calcaneus (Figure 1B). A T-shunt anastomosis was performed between the DCIA and the Posterior Tibial Artery. The skin paddle was sutured in place (Figure 1C). The patient remained non-weight-bearing until pin removal at seven weeks. By two months, she achieved full ambulation and normal footwear use, with no late complications.

### Case 2 – TAP flap

**Figure 2.**
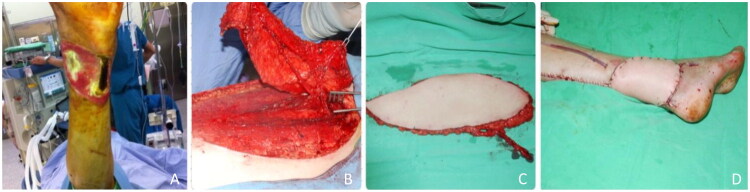
(A) Preoperative picture of the defect. (B) The perforator vessels are isolated. (C) The flap is elevated. (D) Immediate post-operative picture.

A 55-year-old diabetic male developed a chronic heel ulcer with partial necrosis of the Achilles tendon. (Figure 1A). After debridement, a 12 × 6 cm defect remained. A TAP flap (Figure 2B,C) was used for coverage (Figure 2D), with anastomosis to the posterior tibial artery and vein. The patient resumed walking one month postoperatively and achieved full unassisted ambulation by six months, with no recurrence.

### Case 3 – gracilis flap

**Figure 3.**
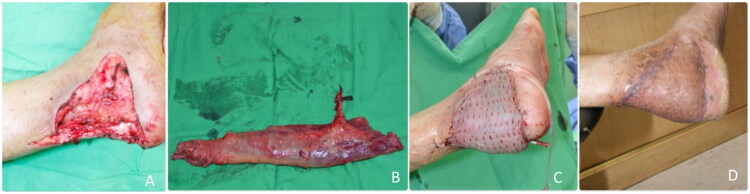
(A) Preoperative picture of the defect. (B) Isolation of the gracilis flap. (C) Immediate post-operative picture. (D) Two-month post-op evaluation.

A 42-year-old male sustained a 4 × 7 cm crush injury to the left foot, without fractures or Achilles tendon involvement. (Figure 3A). A gracilis muscle flap was used, with an end-to-side anastomosis to the posterior tibial artery and skin graft coverage. (Figure 3B-C). The patient resumed normal walking after eight weeks, with sufficient muscle atrophy to accommodate footwear. (Figure 3D).

### Case 4 – forearm flap

**Figure 4.**
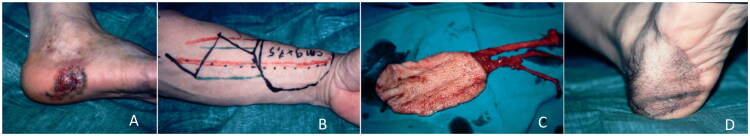
(A) Melanoma located in the right heel. (B) Preoperative planning. (C) the flap is elevated. (D) Eight-months post-operative picture.

A 78-year-old male underwent excision of a 3 × 4 cm melanoma in the right heel (Figure 4A), followed by reconstruction with a free radial forearm flap harvested from the left arm (Figure 4B,C). An end-to-side anastomosis was performed on the posterior tibial artery. At eight months, the flap remained viable, with good color and acceptable scarring. (Figure 4D).

### Case 5 – parascapular flap

**Figure 5.**
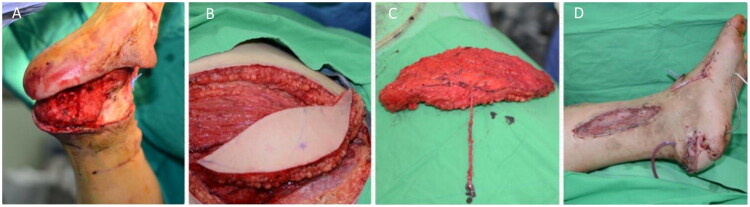
(A) Preoperative picture of the defect. (B) The perforator vessels are isolated and (C) the flap is elevated. (D) Immediate post-operative picture.

A 37-year-old male sustained a left foot crush injury with soft tissue loss, exposing the calcaneus and Achilles tendon (Figure 5A). After debridement, reconstruction was performed using a parascapular flap (Figure 5B,C) with skin grafts for additional coverage (Figure 5D). The flap remained stable postoperatively, requiring only minor graft revision. By two months, the patient regained full ambulation and normal footwear use.

### Case 6 – ALT flap

**Figure 6.**
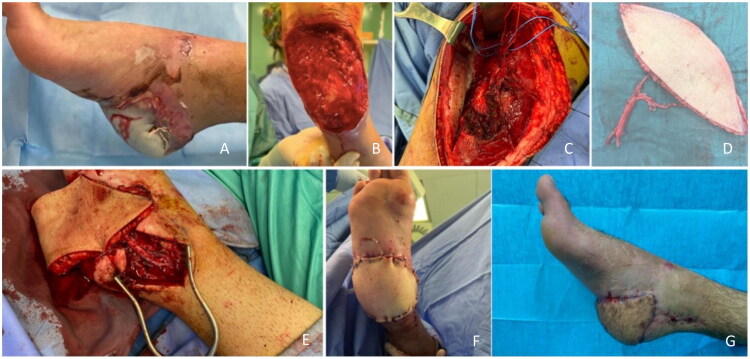
(A) Preoperative picture. (B) Heel after debridement of the necrotic area. (C,D) The perforator vessels are isolated and the flap is elevated. (E) Anastomosis. (F) Immediate post-operative picture. (G) Two- months after the operation.

A 16-year-old male sustained a right heel injury in a motorbike crash, with subsequent soft tissue necrosis (Figure 6A). Extensive debridement was performed (Figure 6B) and an ALT flap from the left thigh was used for reconstruction (Figure 6C,D). Vascular anastomoses involved the posterior tibial artery and concomitant veins (Figure 6E). The flap was secured with a Penrose drain (Figure 6F). Minor post-operative complications (cutaneous mycosis, wound dehiscence) were managed conservatively. After one month of physiotherapy, the patient regained normal ambulation without pain (Figure 6G).

## Discussion

Reconstruction of the calcaneal region requires overcoming challenges related to coverage and functionality. The plantar skin is thick and inelastic, making local flaps inadequate for large or weight-bearing defects. According to literature [1–3,5,8–13] multiple reconstructive options are avaible, and the choice depends on factors such as defect size, involvement of underlying structures (bone, tendons, joints), and local vascular supply. While the instep flap remains the gold standard for small defects reconstruction [9,12,14], the use of free microsurgical flaps is required for reconstruction of extensive defects [9,12,14], especially in post-traumatic patients where the use of loco-regional flaps may be precluded by the trauma itself. In our case series, the flap survival rate was (96%), similar to that reported in other studies [8,9,12,15,16], confirming that free flaps are a viable option for complex heel defect reconstruction. Notably, minor complications were observed in 52% of patients, with excessive bulkiness being the most common issue encountered. Historically muscular flaps have been the gold standard for lower limb reconstruction as several studies reported a superior vascularity, better fracture healing and immunologic properties of such flaps [17]. However, fasciocutaneous flaps have emerged as a valuable alternative due to their adaptability and lower donor-site morbidity [8,13,14,18,19].

In this series, gracilis muscle flaps were primarily used for non-weight-bearing areas where volume replacement was required. Muscle flaps provide three-dimensional coverage, effectively filling voids and adapting to contour changes as the muscle atrophies, facilitating footwear use [9,11,20]. Additionally, they form a stable pad that resists shearing forces and adheres well to the wound bed [21]. As a drawback, their slower sensory recovery often delays weight-bearing and full functional rehabilitation, sometimes necessitating secondary procedures to reduce excessive bulk [11]. For these reasons, we prefer to use muscle flaps to reconstruct defects of the non-weight-bearing areas of the calcaneal region or that partially involve the weight-bearing portion of the heel. The gracilis flap represents a reliable workhorse for reconstructive microsurgery and has a wide reconstructive applicability for both wound coverage and as a functional muscle transplant. Mainly use in diabetic foot reconstruction [20,22] this flap is a valid option for post traumatic heel reconstruction too [10]. As a long and thin muscle, the gracilis flap can be shaped according to the contour of the calcaneal region. The intramuscular neural anatomy allows the muscle to be thinned segmentally, allowing the excessive bulk to be trimmed as needed before the functional muscle is transferred [23]. By opening the epimysium, in fact, it is possible to double or triple its width, tailoring its surface according to the volume of the soft tissue defect [10,20]. For this reason, this flap can be useful even to cover the broader defects. Moreover, its donor site morbidity is low, with no loss of function, and the scar is slightly visible in the upright position, resulting in an excellent aesthetic outcome [24,25]. However, the surgeon must be careful during the dissection to avoid damaging the saphenous nerve [26].As a drawback the short pedicle length make this flap less applicable in post-traumatic patients, as a vascular graft may be needed to reach the recipient vessels on some occasions. The latissimus dorsi free flap might be an alternative option for lower limb reconstruction [27–30]. Due to the long pedicle and the large width of the muscle, this muscle is suitable for extensive defects reconstruction, but it requires to change the patient’s position intraoperatively to harvest the flap most of the time. Nevertheless, even though the donor site morbidity doesn’t have any impact on daily activities, a muscle impairment of the upper limb may hinder the use of crutches during the rehabilitation process needed after lower limb reconstruction, making the use of free gracilis flap a better option for calcaneal region reconstruction.

In our case series nineteen patients (76%) were reconstructed with fasciocutaneous flaps: antero-lateral thigh (ALT) flap was the most used one (eight patients, 32%), followed by thoracodorsal artery perforator (TAP) flap (five cases, 20%), parascapular flap (four patients, 16%) and forearm flap (two cases, 8%). Fasciocutaneous flaps are usually selected following Millard’s principle [31] of replacing tissue with like tissue. Fasciocutaneous flaps are pliable and may be thinned according to the defect conformation adapting the level of flap raising. They all guarantee a low donor site morbidity, with minimal functional sequelae and can resist shear forces [11]. The ALT flap is widely preferred for calcaneal reconstruction due to its reliable anatomy, long pedicle, and low donor-site morbidity [14,32,33]. Its versatility allows for three-dimensional reconstruction [34], making it particularly useful in complex defects requiring volume replacement and tendon coverage [35,36]. A major disadvantage of the ALT flap is that most of the time the thickness of the subcutaneous tissue leads to an excessive bulk. Six out of seven of our patients, in fact, presented this kind of issue. Secondary debulking procedure was performed in all the patients between 6 and 12 months postoperative, with 100% of the patient returning to wear normal shoes afterwards. All these patients experienced also the sliding effect in addition to an excessive bulk: after free flap reconstruction, the dispersion of shear forces through the normal subcutaneous tissue of the heel is lost, resulting in sliding of the foot over the flap while the patients walk [11]. Secondary debulking procedure was efficient in solving this complication too, with all patients returning to normal walking soon after the revision procedure. Another common option for calcaneal region reconstruction is the radial forearm flap. It is a thin and pliable flap that usually doesn’t require a secondary debulking procedure. The surgical anatomy is constant, and a long pedicle with a large caliber is almost always available. Additional advantages include abundant skin paddle dimensions, ease of harvest, and the ability to provide double venous drainage through the paired venae comitantes [37]. Nevertheless, it has a smaller skin paddle and a worst aesthetic result of the donor site in comparison to the ALT flap. For these reasons, it represents a secondary option in our Unit, and it is usually offered to patients whenever the ALT flap cannot be harvested due to trauma or patient’s preference. Together with the radial forearm flap, other fasciocutaneous flaps can be implied in heel reconstruction, are TAP flap [38,39] and parascapular free flap [40]. The flaps based on the subscapular vessel axis may be harvested as chimeric flaps, with different tissue components that can be easily adapted according to the defect. In comparison to the ALT flap, the TAP flap is more pliable and has a thinner subcutaneous tissue and deep fascia, with a subsequent lower risk of excessive bulk and sliding effect. Both TAP and parascapular flap have the advantage to be hairless even in most men but need to be harvested in the lateral or supine position, with the subsequent discomfort of a possible change of the patient’s position during surgery. As for the forearm flap, we opt for these flaps whenever the ALT flap cannot be harvested due to trauma or patient’s preference. In contrast to muscular flap, there is in fact a greater variability in donor sites for fasciocutaneous flap and the location is usually selected in conjunction with the patient. None of the fasciocutaneous flap used in our case series was sensate, as recent studies reported that neurosensorial flaps are not superior in terms of long-term loading capacity and gait functionality compared to non-innervate flaps [9,11,19]. Some form of protective sensation, which is always present in non-sensate fasciocutaneous flaps, seems in fact to be sufficient to provide a durable flap [11].

One patient presented a combined soft tissue and bone defect of the weight-bearing area after a trauma. The management of such defects is a challenging procedure as it requires a composite tissue reconstruction. A single-stage reconstruction is usually preferred by most Authors [41–43] as a two-stage reconstruction is associated with an increased scarring and lack of available recipient vessels [30]. The most used flaps for composite defect reconstruction are the iliac osteocutaneous flap [44,45], the fibula osteoseptocutaneous flap [46], and the muscle-rib flap [47,48]. The vascularized iliac bone is considered the most suitable among these vascularized bone grafts as it is composed by a flat bone that is able to bare the weight of the body [44] and its curvature resembles the calcaneal tuberosity [45]. Even though this flap may not represent the perfect choice for reconstruction of other anatomical areas, in case of heel reconstruction it usually perfectly fits to cover the loss of substance. The vascularized iliac bone is suitable to restore the average anatomical calcaneal dimensions, as it can provide up to 12 cm length and 4 cm of width of bone [44,45]. The main disadvantage of this flap is represented by the high incidence of donor site morbidity, such as gait disturbance and postoperative pain and hematoma; however, the patient included in our case series had no complications in both donor and recipient sites. The fibula osteoseptocutaneous flap may be an alternative bone-containing free flap, but we prefer not to use this flap in post-traumatic patient in order to avoid any adjunctive iatrogenic fracture of the lower limbs. Moreover, for wider defects, the fibula needs to be shaped with and osteotomy and a subsequent osteosynthesis according to the defect [46].

Another important aspect in foot reconstruction is related to the type of anastomosis and the recipient vessels selection [37]. In twenty-four of our cases, we performed an end-to-side (ETS) arterial anastomosis. The advantages of ETS anastomosis include the preservation of the peripheral circulation along with the chance to cope with vessel size discrepancy [49–51]. This is particularly important in post-traumatic patients as it prevents arterial spasm and venous congestion [52–54]. A flow-through anastomosis was performed instead with the iliac crest flap as the patients presented a vascular damaged. This kind of anastomosis allowed us to both reconstruct the posterior arterial artery that was damaged during the trauma, along with the bone and soft tissue defect. Following the principle described by H. C. Chen et al. [55], in case all the vascular axis of the leg are intact, our first choice for recipient vessel is the posterior tibial artery. Less frequently we perform the anastomosis directly on the medial plantar artery, whenever it is intact, close to the defect and matches the size of the donor vessel, in order to preserve the peripheral circulation.

In our case series functional recovery was optimal in all the patients except the one with free flap failure. All the patients were able to walk without any support by 6–12 months post operatively (median period 8 months). The patients who underwent secondary debulking surgery were able to walk again without any support by three months after the revision procedure. Twenty-three out of twenty-four patients (96%) were able to wear normal shoes. The patient with free flap failure subsequently underwent reconstruction with dermal matrix plus skin graft that allows the patient to walk using a crutch. Additionally, donor site morbidity was carefully evaluated. ALT flap harvest was associated with low morbidity, though larger harvests occasionally required skin grafts. Parascapular and TAP flaps showed minimal morbidity, with only minor contour changes noted. Managing donor site morbidity is a key aspect of flap selection, as it impacts overall patient recovery and comfort. For instance, minimizing pain and improving healing at donor sites is crucial, especially in procedures that require substantial tissue harvests. Studies, such as that by Cigna et al. [56], have shown that polyurethane dressings with ibuprofen can be effective in improving pain management and healing outcomes at donor sites for split-thickness skin grafts, suggesting that similar strategies could enhance recovery following flap harvests for heel reconstruction. In contrast, the DCIA flap, used in composite defects, presented higher risks, including potential gait disturbances and pain. These factors were closely monitored, and any complications were managed to ensure optimal recovery for donor sites.

**Figure 7.**
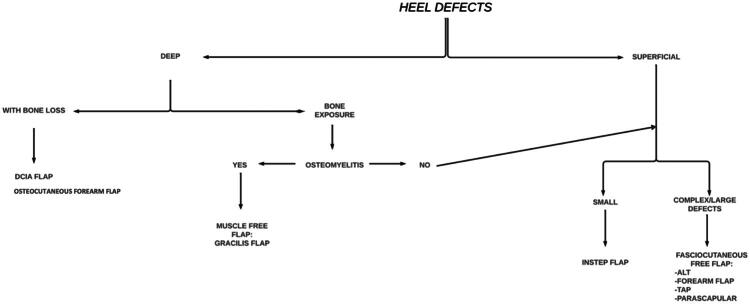
Algorithm for heel reconstruction. ALT flap, anterolateral tight flap; DCIA flap, deep circumflex iliac artery flap; TAP flap, thoracodorsal artery perforator flap.

In response to the complexities of reconstructing the heel region and based on our experience and existing literature [8], we developed a decision-making algorithm (Figure 7) to guide the selection of the most appropriate flap based on defect characteristics, depth, bone involvement, and the presence of infections such as osteomyelitis. This algorithm provides a systematic approach to flap selection and optimizes outcomes for both function and healing quality.

The algorithm directs flap choice as follows: for deep defects with bone loss, the DCIA flap with bone provides essential structural support for weight-bearing defects. However, alternative osteocutaneous flaps, such as the fibula osteoseptocutaneous flap, may also be considered depending on defect characteristics and patient-specific factors, such as the extent of bone loss, vascular integrity of the lower limb, and overall functional demands of the patient. This option is particularly useful when a thin, pliable cutaneous component is needed along with bone reconstruction. In cases of deep defects with bone exposure and osteomyelitis, a muscle free flap, such as the gracilis, is advised in combination with a skin graft. Muscle flaps offer three-dimensional coverage and reduce infection recurrence risk due to their high vascularity. For deep defects with bone exposure but without osteomyelitis, the algorithm suggests treating the defect as a superficial defect, as a muscle flap is unnecessary.

For superficial defects, our approach varies by defect size and complexity. Small defects are managed with an instep flap, which offers adequate coverage while preserving the area’s functional integrity. Complex or large superficial defects, on the other hand, require fasciocutaneous flaps such as ALT or TAP, which are durable and provide broad coverage for mechanically stressed regions.

Over time, we developed this decision-making algorithm based on the experience gained from our case series and insights from existing literature, enabling a more systematic approach tailored to the specific requirements of each defect. This algorithm therefore offers a practical framework that may assist other plastic surgeons in selecting the most suitable flap, ensuring optimal functional and aesthetic results. Our experience suggests that a structured, defect-based approach aids in making more effective surgical decisions, contributing to more predictable healing outcomes.

The study boasts significant strengths, including its extensive span across two decades, which allowed for a comprehensive analysis of the evolution of surgical techniques and outcomes in heel reconstruction. The diverse array of free flaps used in this case series reflects the complexity and variability encountered in clinical practice, offering valuable insights into flap selection strategies tailored to specific defect characteristics. The notable success rate of flap survival, along with meticulously documented complications and their management, enhances the current body of knowledge and serves as a useful resource for surgeons. Importantly, the study’s focus on functional recovery underscores its commitment to patient-centered care, demonstrating the positive impact of microsurgical reconstruction on enhancing patients’ quality of life. While this retrospective study provides meaningful findings, inherent limitations such as selection bias and challenges in controlling all outcome-affecting variables are acknowledged. Additionally, the broad spectrum of defect etiologies and reconstruction techniques, though demonstrating versatility, complicates direct outcome comparisons. The relatively modest sample size and absence of a control group also limit the broader applicability of the findings. Furthermore, the average follow-up duration of eight months may not fully capture long-term complications or the durability of functional improvements. Nonetheless, our experience, combined with existing literature, has allowed us to develop a decision-making algorithm for flap selection. This algorithm provides a structured approach that may assist surgeons in choosing the most appropriate flap based on defect characteristics, contributing to systematic improvements in outcomes for heel reconstruction.

## Study limitations

This study has several limitations. The small sample size (25 patients) limits the generalizability of our findings, as a larger cohort would provide more statistically robust conclusions. Additionally, the retrospective design and the absence of a control group prevent direct comparisons with other reconstructive techniques, such as local flaps or alternative microsurgical approaches. Another significant limitation is the follow-up period, which had a median duration of 8 months. While this timeframe primarily focuses on early functional recovery, it aligns with the critical period for evaluating surgical success, as most complications in free flap reconstruction tend to occur within the first months postoperatively. Moreover, all patients who achieved stable ambulation and footwear adaptability at 8 months showed no signs of flap failure, suggesting that major long-term complications are unlikely in this cohort.

## Conclusions

This study validates microsurgical free flap reconstruction as an effective strategy for complex heel defects, highlighting a 96% flap survival rate and significant functional recovery, including walking and footwear adaptability. Despite challenges like flap bulkiness necessitating revision, the overall outcomes underscore microsurgery’s pivotal role in enhancing quality of life for patients with calcaneal injuries. It affirms the necessity of personalized, advanced surgical approaches in optimizing lower extremity reconstruction [2,3,8,14,19].

## Data Availability

The data used and/or analyzed during the current study are available from the corresponding author upon reasonable request.
